# Supplementary material for: Cryo-electron tomography provides topological insights into mutant huntingtin exon 1 and polyQ aggregates
Source: Commun Biol. 2021 Jul 8;4:849. doi: 10.1038/s42003-021-02360-2 (PMC8266869; doi:10.1038/s42003-021-02360-2)
Supplement: Supplementary file 2 — Supplementary Information [file 42003_2021_2360_MOESM2_ESM.pdf]

# Cryo-Electron Tomography Provides Topological Insights into Mutant Huntingtin Exon 1 and PolyQ Aggregates

Jesús G. Galaz-Montoya[1‡], Sarah H. Shahmoradian[2*], Koning Shen[3*†], Judith Frydman[3], Wah Chiu[1,4‡]

[1] Department of Bioengineering and James H. Clark Center, Stanford University, Stanford, CA 94305, United States of America

[2] Department of Biology and Chemistry, Laboratory of Biomolecular Research, Paul Scherrer Institute, Villigen, Switzerland

[3] Department of Biology, Stanford University, Stanford, CA 94305, United States of America

[4] Division of CryoEM and Bioimaging, SSRL, SLAC National Accelerator Laboratory, Menlo Park, CA 94025, United States of America

* Equal contribution

† Current address: Department of Molecular and Cell biology, University of California, Berkeley, CA 94720, United States

‡ Correspondence to: Jesús G. Galaz-Montoya, e-mail: jgalaz@gmail.com; Wah Chiu, e-mail: wahc@stanford.edu

# SUPPLEMENTARY INFORMATION

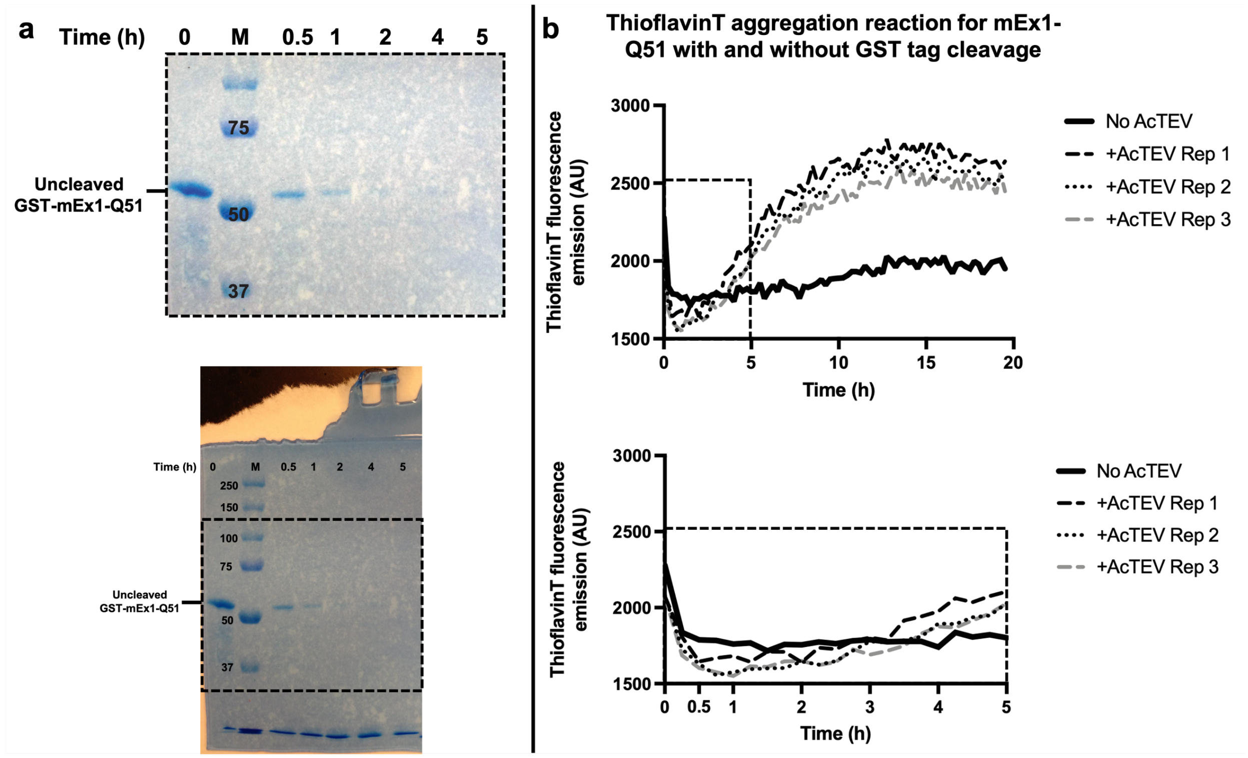


**Supplementary Figure 1. Purification, cleavage, and aggregation of GST-mEx1-Q51 with and without GST tag cleavage. (a)** SDS-PAGE gel stained with Coomassie showing AcTEV cleavage of GST-mEx1-Q51 over time, with 0 h indicating intact protein before addition of AcTEV protease (Invitrogen) and "M" indicating protein ladder (BioRad) with relevant molecular weights listed in kDa. **(b)** ThioflavinT aggregation reaction (top) of GST-mEx1-Q51 including no-AcTEV control (no aggregation) and three technical replicates with AcTEV added (aggregation reaction), and close-up of first five hours of aggregation reaction (bottom), showing minimal aggregation before 2 h (i.e., all aggregation happens post-AcTEV cleavage).

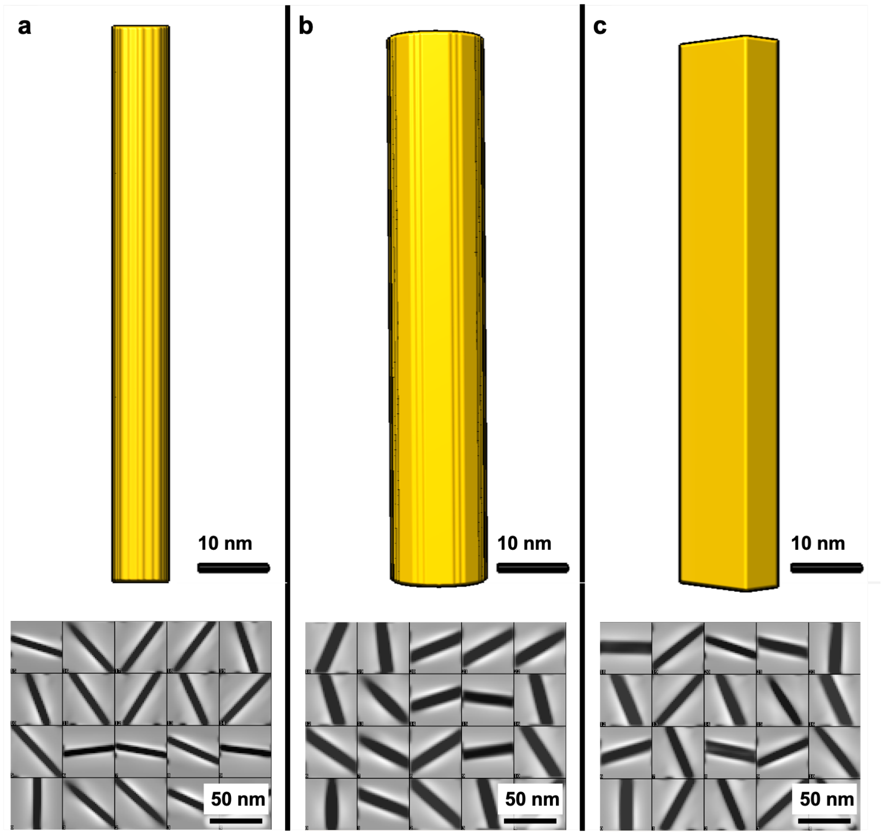


**Supplementary Figure 2. Slab-shaped filaments are more consistent with observations of variable width in central Z cross-sections (in the XY plane, unaffected by the missing wedge artifact) than cylindrical filaments.** Simulated model and corresponding central Z cross-sections of simulated subtomograms for a cylinder **(a)** 7 nm or **(b)** 15 nm in diameter, and **(c)** a rectangular slab with narrow and wide sides measuring 7 and 15 nm, respectively. Scale bars: 10 nm next to the 3D models, and 50 nm on top of the 2D slices through simulated subtomograms.

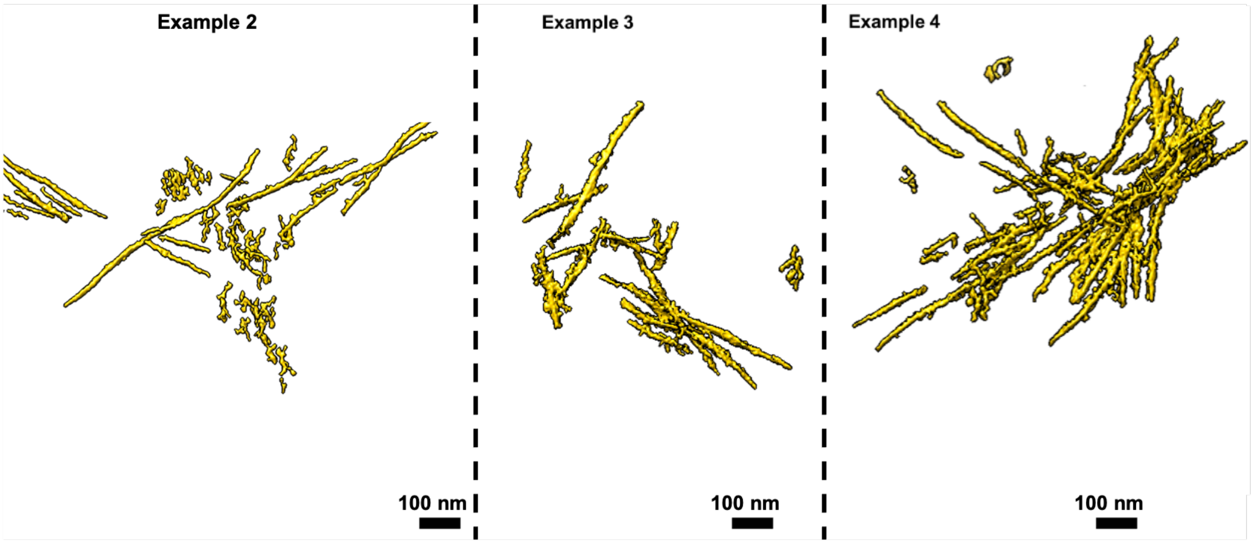


**Supplementary Figure 3.** Additional examples of annotated mEx1-Q51 filamentous aggregates in cryoET tomograms, annotated semi-automatically using neural networks. The smaller densities seen in regions of examples 2 and 4 may correspond to oligomers or protofilaments. Scale bars: 100 nm.

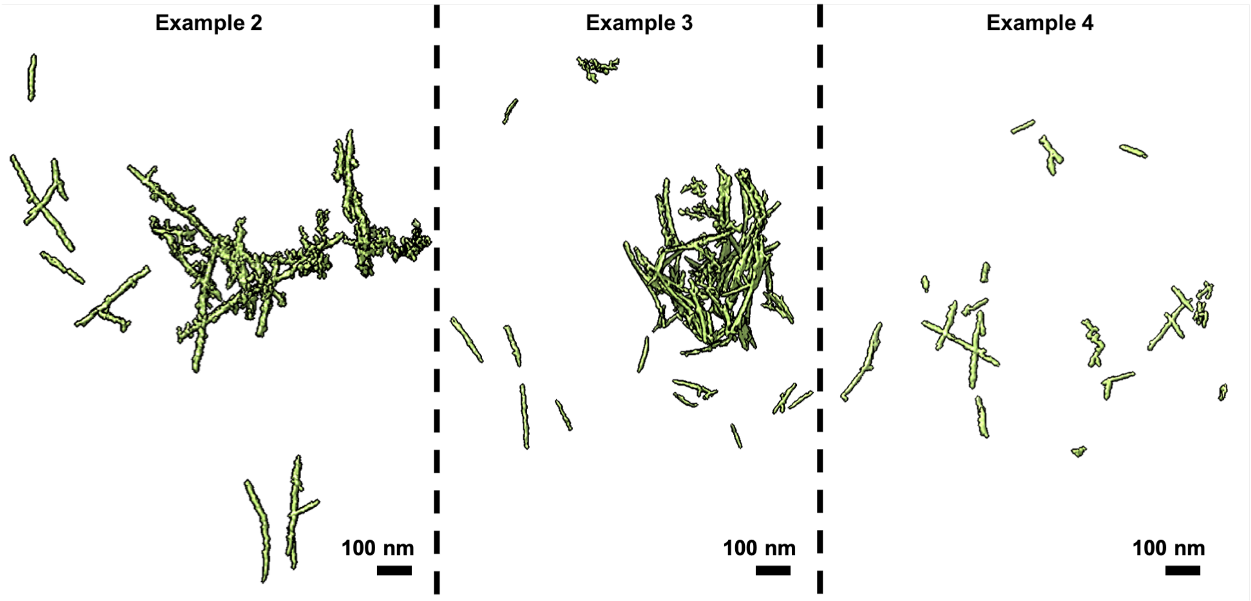


**Supplementary Figure 4.** Additional examples of annotated Q51 filamentous aggregates in cryoET tomograms, annotated semi-automatically using neural networks. The smaller densities seen in regions of examples 3 and 4 may correspond to oligomers or protofilaments. Scale bars: 100 nm.

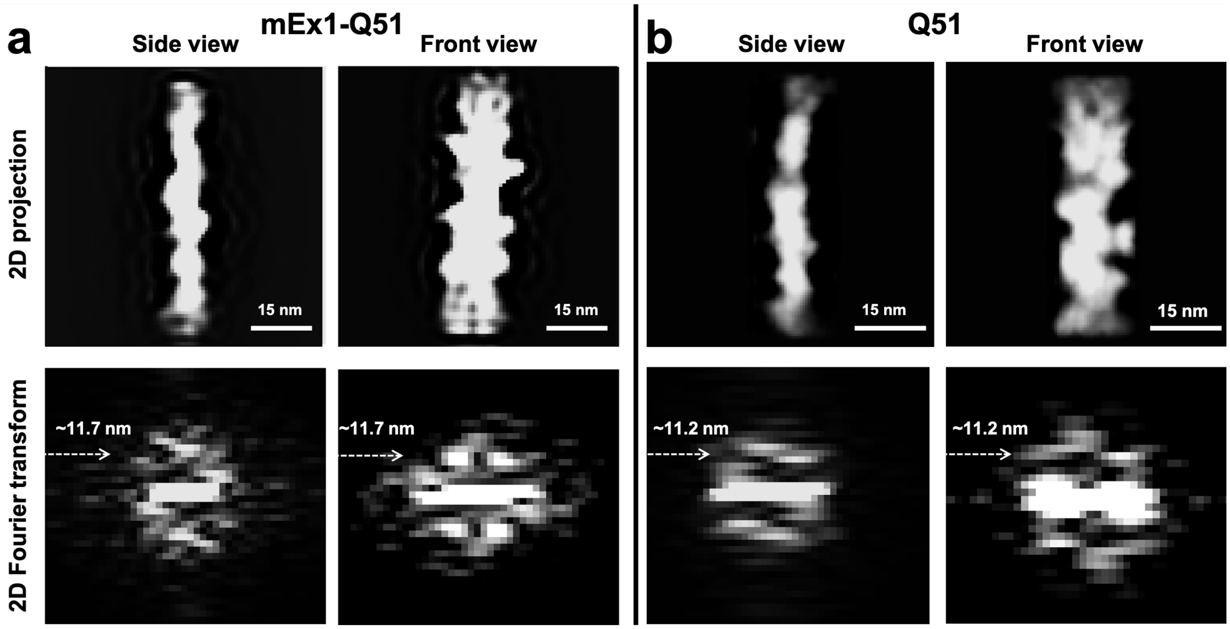


**Supplementary Figure 5.** Orthogonal side and face-on projections from the subtomogram averages of **(a)** mEx1-Q51 (Figure 2C) and **(b)** Q51 (Figure 4C) filaments, and their corresponding power spectra showing bright maxima off of the meridian in both cases at ~11.2-11.7 nm, suggestive of pseudo-periodicity in the subpopulation of filament segments that contributed to the subtomogram averages. Scale bars: 15 nm.
