# Supplementary material for: Cryo-electron tomography provides topological insights into mutant huntingtin exon 1 and polyQ aggregates
Source: Commun Biol. 2021 Jul 8;4:849. doi: 10.1038/s42003-021-02360-2 (PMC8266869; doi:10.1038/s42003-021-02360-2)
Supplement: Supplementary file 3 — Description of Additional Supplementary Files [file 42003_2021_2360_MOESM3_ESM.pdf]

Description of Additional Supplementary Files

File name: Supplementary Data 1

Description: Excel file containing the raw data used to generate the plots in Supplementary Figure 1b, showing ThioflavinT aggregation reaction of GST-mEx1-Q51 including no-AcTEV control (no aggregation) and three technical replicates with AcTEV added (aggregation reaction).
