# Supplementary material for: Cryo-electron tomography provides topological insights into mutant huntingtin exon 1 and polyQ aggregates
Source: Commun Biol. 2021 Jul 8;4:849. doi: 10.1038/s42003-021-02360-2 (PMC8266869; doi:10.1038/s42003-021-02360-2)
Supplement: Supplementary file 5 — Reporting Summary [file 42003_2021_2360_MOESM5_ESM.pdf]

nature research

Corresponding author(s): Galaz-Montoya Jesus G., Chiu W.

Last updated by author(s): 5/12/2021

Reporting Summary

Nature Research wishes to improve the reproducibility of the work that we publish. This form provides structure for consistency and transparency in reporting. For further information on Nature Research policies, see our Editorial Policies and the Editorial Policy Checklist.

Statistics

For all statistical analyses, confirm that the following items are present in the figure legend, table legend, main text, or Methods section.

n/a	Confirmed	
☐	☒	The exact sample size (n) for each experimental group/condition, given as a discrete number and unit of measurement
☐	☒	A statement on whether measurements were taken from distinct samples or whether the same sample was measured repeatedly
☒	☐	The statistical test(s) used AND whether they are one- or two-sided<br>*Only common tests should be described solely by name; describe more complex techniques in the Methods section.*
☒	☐	A description of all covariates tested
☒	☐	A description of any assumptions or corrections, such as tests of normality and adjustment for multiple comparisons
☒	☐	A full description of the statistical parameters including central tendency (e.g. means) or other basic estimates (e.g. regression coefficient) AND variation (e.g. standard deviation) or associated estimates of uncertainty (e.g. confidence intervals)
☒	☐	For null hypothesis testing, the test statistic (e.g. F, t, r) with confidence intervals, effect sizes, degrees of freedom and P value noted<br>*Give P values as exact values whenever suitable.*
☒	☐	For Bayesian analysis, information on the choice of priors and Markov chain Monte Carlo settings
☒	☐	For hierarchical and complex designs, identification of the appropriate level for tests and full reporting of outcomes
☒	☐	Estimates of effect sizes (e.g. Cohen's d, Pearson's r), indicating how they were calculated

Our web collection on statistics for biologists contains articles on many of the points above.

Software and code

Policy information about availability of computer code

Data collection	Serial EM 3.0 was used for data collection.
Data analysis	IMOD 4.9, ICON-GPU 1.2.9, and EMAN 2.3 were used for tomographic alignment and reconstruction and the latter also for subtomogram averaging, and UCSF Chimera 1.14 was used for visualization.

For manuscripts utilizing custom algorithms or software that are central to the research but not yet described in published literature, software must be made available to editors and reviewers. We strongly encourage code deposition in a community repository (e.g. GitHub). See the Nature Research guidelines for submitting code & software for further information.

Data

Policy information about availability of data

All manuscripts must include a data availability statement. This statement should provide the following information, where applicable:

- Accession codes, unique identifiers, or web links for publicly available datasets
- A list of figures that have associated raw data
- A description of any restrictions on data availability

The raw data can be made accessible upon request. The Electron Microscopy Data Bank accession numbers for the structures reported in this paper are as follows: mEx1-Q51 subtomogram average, EMD-21248; Q51 subtomogram average, EMD-21253.

Field-specific reporting

Please select the one below that is the best fit for your research. If you are not sure, read the appropriate sections before making your selection.

- [x] Life sciences
- [ ] Behavioural & social sciences
- [ ] Ecological, evolutionary & environmental sciences

For a reference copy of the document with all sections, see nature.com/documents/nr-reporting-summary-flat.pdf

Life sciences study design

All studies must disclose on these points even when the disclosure is negative.

Sample size	No sample size calculation was made a priori. We froze and screened multiple cryoEM grids with specimen, collected ~20+ tiltseries from those that were suitable for cryoET imaging for mEx1-Q51 aggregates, and analyzed aggregated filaments in the reconstructed tomograms that contained large aggregates (n=6). The Q51 aggregate data were collected later and the target was to achieve a comparable sample size as for mEx1-Q51 (correspondingly, we also analyzed Q51 aggregated filaments from n=6 tomograms).
Data exclusions	Tilt series exhibiting too many artifacts such as extensive radiation damage, excessive drift, charging, missing images, etc., were not processed. For some tilt series, images with artifacts were manually excluded from tomographic alignment and reconstruction. Among the reconstructed tomograms, only those with large filament aggregates not on the carbon film but rather in the aqueous meniscus in the grid hole were subjected to downstream analyses such as neural net semi-automated annotation and subtomogram averaging (STA). For STA, low-correlating particles were excluded through iterative refinement (~50% of all extracted subtomograms were kept in the final subtomogram averages).
Replication	We have extensive experience preparing mEx1-Q51 and Q51 aggregates and those of related constructs and screening them with cryo electron microscopy (cryoEM). The preparation of mEx1-Qn and Qn aggregates on cryoEM grids is reproducible. See Shahmoradian et al. 2013, Darrow et al. 2015, Shen et al. 2016 cited in the References, as well as multiple references from Judith Frydman's lab describing mHTT aggregation through nearly two decades. For the cryoET experiments presented here, the data come from multiple cryoEM grids and experiments, which yielded multiple tomograms with consistent features, attesting to the reproducibility of the tomograms. We carried out annotation semi-automatically using neural networks, without the use of templates. The method is documented (Chen 2017, Nature Methods) and reproducible. Importantly, we trained a separate neural network for each tomogram, sometimes from different versions of the tomogram reconstructed with IMOD, ICON-GPU, and EMAN2; the annotations are comparable and reproducible. For subtomogram averaging, we followed best practices by carrying out gold-standard, iterative refinement without the use of external models or templates, using particles from multiple tomograms.
Randomization	N/A.
Blinding	N/A.

Reporting for specific materials, systems and methods

We require information from authors about some types of materials, experimental systems and methods used in many studies. Here, indicate whether each material, system or method listed is relevant to your study. If you are not sure if a list item applies to your research, read the appropriate section before selecting a response.

Materials & experimental systems

n/a	Involved in the study
☒	☐ Antibodies
☒	☐ Eukaryotic cell lines
☒	☐ Palaeontology and archaeology
☒	☐ Animals and other organisms
☒	☐ Human research participants
☒	☐ Clinical data
☒	☐ Dual use research of concern

Methods

n/a	Involved in the study
☒	☐ ChIP-seq
☒	☐ Flow cytometry
☒	☐ MRI-based neuroimaging
